# HALP, a routine nutrition-inflammation index, and mortality across the cMetS spectrum: NHANES with supportive external cohort evidence

**DOI:** 10.3389/fnut.2026.1818651

**Published:** 2026-05-20

**Authors:** Keting Deng, Yang Yao, Biping Cheng, Xuan Wang

**Affiliations:** 1 Department of Clinical Laboratory, The First Affiliated Hospital of Xi’an Medical University, Xi’an, Shaanxi, China; 2 Department of Critical Care Medicine, The First Affiliated Hospital of Xi’an Medical University, Xi’an, Shaanxi, China; 3 Department of Functional Examination, The First Affiliated Hospital of Xi’an Medical University, Xi’an, Shaanxi, China; 4 Department of Cardiovascular Medicine (VIP Ward), Yan’an University Xianyang Hospital, Xianyang, Shaanxi, China

**Keywords:** all-cause mortality, cardiovascular mortality, continuous metabolic syndrome score, hemoglobin-albumin-lymphocyte-platelet index, metabolic burden

## Abstract

**Background:**

The continuous metabolic syndrome score (cMetS) quantifies cardiometabolic burden but may not fully capture systemic vulnerability related to nutrition, immunity, and thrombo-inflammation. We examined the prognostic value of the hemoglobin-albumin-lymphocyte-platelet (HALP) index across the cMetS spectrum.

**Methods:**

Adults aged ≧ 20 years from NHANES 1999–2010 were followed through 31 December 2019. HALP was assessed using survey-weighted Cox regression with restricted cubic splines, adjusting for cMetS and covariates. Additional analyses included absolute risk, joint HALP-cMetS classification, Harrell’s C statistic, decision curve analysis, competing-risk models, and supportive external cohort analysis.

**Results:**

Among 10,770 participants, lower HALP was associated with higher all-cause mortality. Compared with the lowest HALP tertile, adjusted HRs were 0.78 (95% CI, 0.69–0.88) for the middle tertile and 0.86 (95% CI, 0.77–0.95) for the highest tertile. Ten-year mortality risk was highest in the lowest HALP tertile: 15.4% versus 11.8 and 12.2% in the middle and highest tertiles. The association was nonlinear and L-shaped. Participants with high cMetS and low HALP had the highest risks of all-cause mortality (HR, 1.32, 95% CI, 1.15–1.52) and cardiovascular mortality (HR, 1.56,95% CI, 1.16–2.09). Adding HALP modestly improved discrimination beyond cMetS and covariates (Harrell’s C, 0.8666 to 0.8685; ΔC = 0.0018), with modest net benefit. Competing-risk analyses attenuated cardiovascular mortality associations. A supportive external cohort analysis supported the all-cause mortality association but was limited for cardiovascular mortality.

**Conclusion:**

HALP was independently and nonlinearly associated with mortality across the cMetS spectrum and provided modest prognostic information beyond cMetS. As an inexpensive routine index, HALP may complement metabolic burden assessment by identifying systemic vulnerability.

## 1 Introduction

Metabolic syndrome is highly prevalent worldwide and is associated with increased risks of all-cause and cardiovascular mortality (1). Traditional clinical definitions of metabolic syndrome are dichotomous and threshold based. Although useful for diagnosis, such definitions do not fully reflect the graded and continuous nature of metabolic abnormalities. Increasing epidemiological evidence suggests that cardiometabolic risk operates along a continuum, with progressively higher metabolic burden associated with incrementally greater risk of adverse outcomes (2). To address this limitation, the continuous metabolic syndrome score (cMetS) has been developed to integrate multiple metabolic components into a single quantitative metric (3). By capturing continuous variation in waist circumference, blood pressure, lipid levels, and glucose metabolism, cMetS provides a more refined assessment of metabolic dysregulation and facilitates evaluation of graded associations in population studies (4). However, even among individuals with similar cMetS levels, prognosis varies considerably. This residual heterogeneity suggests that systemic factors beyond conventional metabolic burden contribute meaningfully to individual risk. Emerging evidence indicates that chronic low grade inflammation, nutritional status, immune function, and hematologic reserve are closely linked to cardiometabolic progression and mortality (5–7). These systemic dimensions are not captured by standard metabolic measures. Therefore, identifying easily obtainable biomarkers that reflect these systemic dimensions may improve risk assessment across the spectrum of metabolic health.

The hemoglobin, albumin, lymphocyte, and platelet (HALP) index is a composite biomarker derived from routine laboratory tests. It integrates indices of oxygen carrying capacity, nutritional and inflammatory status, immune competence, and thrombo inflammatory activity (8). Although HALP is not yet a widely established clinical risk score, it is inexpensive, routinely available, and biologically integrative, which makes it attractive for population-based risk assessment research. HALP has been increasingly investigated in cardiovascular and metabolic populations. Studies have reported that lower HALP levels are associated with higher risks of all cause and CVD mortality in patients with coronary heart disease and acute myocardial infarction (9, 10). Yet its prognostic value in broader adult populations, and its relationship with continuously quantified metabolic burden, remains unclear. Two questions are particularly relevant: whether HALP provides prognostic value beyond cMetS, and whether combining HALP with metabolic burden improves identification of individuals at elevated risk.

To answer these questions, we conducted a cohort study using nationally representative data from United States adults in NHANES from 1999 to 2010 with linked mortality follow up, complemented by external validation in an independent hospital based cohort. We evaluated the association between HALP and all cause and CVD mortality, examined potential nonlinear dose–response relationships, and assessed whether associations differed across levels of metabolic burden quantified by cMetS. These findings may clarify whether a simple and routinely available composite laboratory index can capture systemic vulnerability beyond metabolic burden alone and thereby support characterization of prognostic heterogeneity across the cardiometabolic spectrum.

## 2 Methods

### 2.1 Study design and population

The National Health and Nutrition Examination Survey is an ongoing, nationally representative program designed to assess the health and nutritional status of the noninstitutionalized civilian population of the United States. NHANES employs a complex, multistage probability sampling design and collects data through standardized interviews, physical examinations, and laboratory measurements (11). Survey protocols were approved by the National Center for Health Statistics Research Ethics Review Board, and all participants provided written informed consent.

The present study included participants from NHANES 1999 to 2010 who were aged 20 years or older. Of 87,982 participants initially screened, 28,615 individuals younger than 20 years were excluded. Among the remaining 59,367 adults, 48,511 were excluded because of incomplete data required to calculate HALP, derive cMetS, or adjust for covariates in the fully adjusted models. Among the 10,856 participants with complete baseline data, we further excluded 81 participants with follow-up < 12 months and 5 without mortality information. The final analytic sample included 10,770 participants.

### 2.2 Definitions of exposure and outcome variables

#### 2.2.1 Exposure variables

Metabolic burden was assessed using the cMetS. The score was constructed by integrating five core metabolic components measured at baseline: waist circumference, triglycerides, high-density lipoprotein cholesterol, mean arterial pressure(MAP), and fasting glucose. MAP was calculated from the average of up to three systolic (SBP) and diastolic (DBP) readings using the standard formula: MAP=DBP + (SBP - DBP)/3, consistent with prior cMetS derivations (12). Each component was transformed into a standardized z score before deriving cMetS, and the score was calculated as the sum of standardized metabolic components (13).


cMetS=z(WC)+z[ln(TG)]+z(MAP)+z[ln(glucose)]−z(HDL−C).

Where z represents the standardized value of each metabolic component.

Systemic vulnerability was assessed using the hemoglobin, albumin, lymphocyte, and platelet (HALP) index, calculated from baseline laboratory measurements as (14).


$$\text{HALP}=\frac{\text{hemoglobin}\times \text{albumin}\times \text{lymphocyte count}}{\text{platelet count}}$$

#### 2.2.2 Outcome variables

The outcome variables included all-cause mortality and CVD mortality. Mortality data for the follow up population were obtained from the NHANES public use linked mortality files, with updates through December 31, 2019 (15). These records were matched to the National Center for Health Statistics and the National Death Index using probabilistic linkage algorithms. Mortality outcomes were coded according to the International Classification of Diseases, Tenth Revision.

The observation time was defined as the interval between the baseline assessment and the occurrence of death or the end of follow up, whichever came first. All-cause mortality included deaths from any cause, such as heart disease, malignant neoplasms, unintentional injuries, cerebrovascular diseases, diabetes mellitus, and other causes. CVD mortality referred specifically to deaths attributed to heart disease or cerebrovascular diseases, defined using ICD 10 codes I00 to I09, I11, I13, I20 to I51, and I60 to I69 (16).

### 2.3 Covariates

Covariates were selected *a priori* based on clinical relevance and prior literature and were obtained from baseline questionnaires, examinations, and laboratory measurements. Age was modeled as a continuous variable in years. Sex was categorized as male or female. Race and ethnicity was categorized as non Hispanic White, non Hispanic Black, Mexican American, and other race including multiracial. Education level was categorized as less than high school, high school graduate or equivalent, and more than high school. Marital status was categorized as married or living with partner, never married, and other. Socioeconomic status was assessed using the poverty income ratio and was included as a continuous variable.

Smoking status was categorized as never, former, or current based on self reported cigarette use. Alcohol consumption was categorized as never, former, or current based on questionnaire responses. Hypertension was defined as mean systolic blood pressure of at least 140 mmHg, mean diastolic blood pressure of at least 90 mmHg, self reported physician diagnosis of hypertension, or current use of antihypertensive medication. Estimated glomerular filtration rate was calculated using the Chronic Kidney Disease Epidemiology Collaboration equation based on serum creatinine and was modeled as a continuous variable (16). Survey cycle was included as a categorical covariate to account for potential temporal variation across survey cycles.

### 2.4 Statistical analysis

#### 2.4.1 NHANES cohort

All analyses followed the NHANES analytic and reporting guidelines and accounted for the complex survey design by incorporating sampling weights, strata, and primary sampling units. For pooled analyses across NHANES 1999–2010, the 2-year fasting subsample weights (WTSAF2YR) were divided by the number of combined cycles (*n* = 6) to create multi-cycle weights. All analyses incorporated the complex survey design (weights, strata, and PSUs). For baseline descriptive analyses, continuous variables were summarized as survey-weighted means with standard errors, and categorical variables were presented as unweighted counts with survey-weighted percentages. Differences across cMetS tertile groups were assessed using design-based tests, including survey-weighted linear regression for continuous variables and Rao-Scott adjusted chi-square tests for categorical variables. Associations between HALP and mortality outcomes were evaluated using survey-weighted Cox proportional hazards regression to estimate hazard ratios (HRs) and 95% confidence intervals (CIs). To improve clinical interpretability, we additionally estimated 10-year absolute risk of all-cause mortality across HALP tertiles using Kaplan–Meier survival estimates. Three sequential models were fitted: Model 1 adjusted for age, sex, and race. Model 2 additionally adjusted for education, marital status, family income–poverty ratio, drinking status, and smoking status. Model 3 further adjusted for hypertension status, estimated glomerular filtration rate (eGFR), and continuous cMetS score. The proportional hazards assumption was assessed using Schoenfeld residuals and graphical methods. Potential nonlinear dose–response relationships were examined using restricted cubic splines with knots placed at the 10th, 50th, and 90th percentiles of the survey-weighted HALP distribution, and overall and nonlinear *p* values were reported.

To assess whether HALP provided incremental prognostic information beyond cMetS, we constructed two nested models based on the fully adjusted covariate set used in the primary analysis. Model A included age, sex, race/ethnicity, education, marital status, poverty-income ratio, smoking status, drinking status, hypertension, estimated glomerular filtration rate, survey cycle, and cMetS. Model B additionally included HALP. Because the primary analyses indicated a nonlinear association between HALP and mortality, HALP was modeled flexibly in the incremental discrimination analysis. Model discrimination was compared using Harrell’s C statistic, and bootstrap resampling was used to estimate the confidence interval for the change in C statistic. Decision curve analysis was performed for 10-year all-cause mortality to assess potential clinical net benefit.

Effect modification by metabolic burden was examined through analyses stratified by cMetS category (highest tertile versus lower two tertiles) and by testing multiplicative interaction via a product term between continuous HALP and cMetS category in the fully adjusted model. Joint exposure categories were defined by HALP (lowest tertile versus upper two tertiles) and cMetS category (highest tertile versus lower two tertiles), using the non-high cMetS and non-low HALP group as the reference. For CVD mortality, the cause-specific Cox model was treated as the primary analysis because it estimates the etiologic association between HALP and the instantaneous hazard of CVD death among participants who remain at risk. Non-cardiovascular death was additionally treated as a competing event in Fine-Gray subdistribution hazard models, which were performed as sensitivity analyses to evaluate the association between HALP and the cumulative incidence of CVD death in the presence of competing non-CVD mortality. Fine-Gray models were adjusted for the covariates in Model 3. Sensitivity analyses excluded participants who died within the first 24 months of follow up to minimize potential reverse causality. All tests were two-tailed, and a p value less than 0.05 was considered statistically significant.

#### 2.4.2 Supportive external cohort analysis

A supportive analysis was performed in an independent cardiology cohort from Yan’an University Xianyang Hospital (*n* = 500; follow up to 36 months; all-cause deaths, *n* = 36; cardiovascular deaths, *n* = 17). The cohort was retrospectively collected between January 2018 and December 2024. The study protocol was approved by the Ethics Committee of Yan’an University Xianyang Hospital (Approval No. YDXY-KY-2025-089). Given the retrospective nature of the study and the use of de-identified data, the requirement for informed consent was waived, and the ethics approval covered the retrospective use of data collected prior to the approval date in accordance with local regulations. Baseline characteristics were summarized using standard descriptive statistics. HALP was calculated using the same formula as in NHANES and categorized into tertiles based on cohort-specific cutpoints. Associations between HALP tertiles and mortality were evaluated using Cox proportional hazards models, reporting HRs and 95% CIs, with adjustment for age, sex, and available covariates corresponding to those included in the NHANES fully adjusted model. All-cause death was ascertained through telephone follow-up and hospital records. Cardiovascular death was classified as death attributed to cardiovascular causes based on hospital records supplemented by telephone follow-up. Formal endpoint adjudication by an independent committee was not performed, and autopsy-based morphological confirmation of cardiovascular death was unavailable. CVD mortality analyses were performed where feasible, and estimates were interpreted cautiously when event numbers were limited. All analyses were conducted using R (version 4.3.0); the survey and survival packages were used for NHANES analyses.

## 3 Results

### 3.1 Study population and baseline characteristics

**Figure 1:**
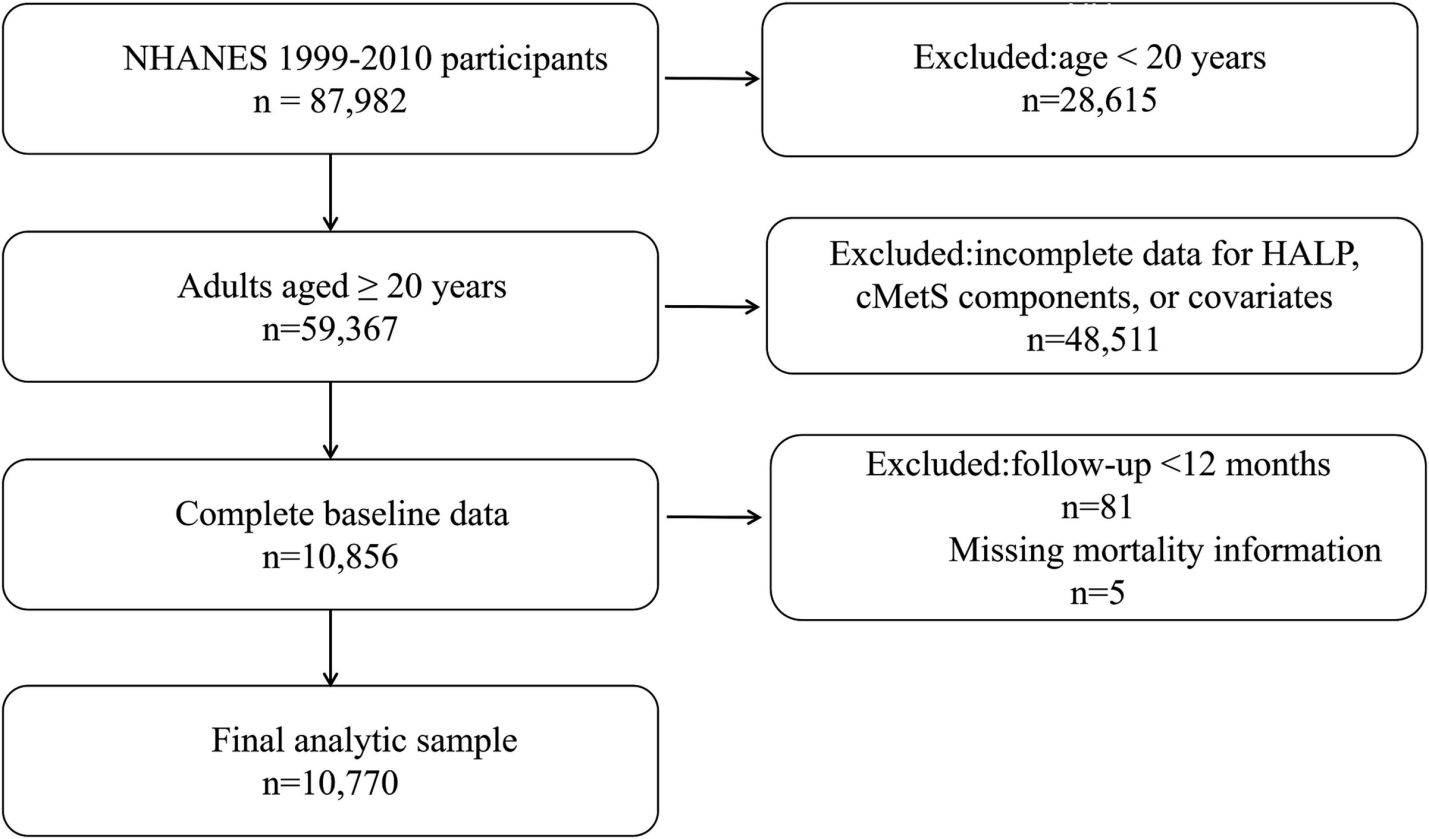
Flowchart of participant selection in NHANES 1999–2010. HALP, hemoglobin-albumin-lymphocyte-platelet index; cMetS, continuous metabolic syndrome score; NHANES, National Health and Nutrition Examination Survey.

Participant selection for the NHANES 1999–2010 analysis is summarized in Figure 1. We first excluded individuals younger than 20 years (*n* = 28,615). Among adults aged ≥ 20 years, participants with missing information required to calculate cMetS or HALP, or with missing key covariates, were excluded (*n* = 48,511), leaving 10,856 participants with complete data. We further excluded participants with follow up time <12 months (*n* = 81) and those without available mortality information (*n* = 5). The final NHANES analytic sample therefore included 10,770 participants.

In the survey-weighted NHANES 1999–2010 analytic sample (*N* = 10,770), participants were categorized into cMetS tertiles using survey-weighted cutpoints. Higher cMetS tertiles were associated with older age and lower eGFR (both *p* < 0.001), whereas the poverty income ratio was comparable across tertiles (*p* = 0.119). The HALP index increased across tertiles (*p* < 0.001). Sociodemographic and behavioral profiles differed by metabolic burden (all *p* < 0.001), including sex distribution, race/ethnicity, education, marital status, smoking, and alcohol consumption. Hypertension prevalence rose markedly from T1 to T3 (17.5% vs. 56.2%). Mortality events were more frequent in higher tertiles, with all-cause death increasing from 9.6 to 21.0% and cardiovascular death from 2.8 to 6.9% (both *p* < 0.001)(Table 1).

**Table 1:** Baseline characteristics of participants according to survey-weighted cMetS tertiles in NHANES 1999–2010.

Characteristic	cMetS T1	cMetS T2	cMetS T3	*P* value
N	3,330	3,589	3,851	
Age, years	41.73 (0.41)	46.94 (0.45)	50.44 (0.32)	<0.001
eGFR, mL/min/1.73m^2^	101.94 (0.54)	97.36 (0.60)	94.38 (0.56)	<0.001
HALP index	48.94 (1.04)	50.22 (0.49)	55.18 (0.68)	<0.001
Poverty income ratio	3.12 (0.04)	3.09 (0.05)	3.03 (0.04)	0.119
Sex				<0.001
Male	1,059 (30.8)	1810 (51.3)	2,441 (65.8)	
Female	2,271 (69.2)	1779 (48.7)	1,410 (34.2)	
Race				<0.001
Mexican American	518 (6.2)	737 (8.0)	898 (8.1)	
Non-Hispanic Black	676 (11.2)	623 (9.4)	609 (8.4)	
Non-Hispanic White	1788 (73.0)	1865 (73.1)	1992 (74.4)	
Others	348 (9.6)	364 (9.5)	352 (9.1)	
Education				<0.001
< High school	736 (14.5)	1,043 (18.6)	1,305 (21.7)	
High school	719 (22.0)	850 (25.2)	996 (29.1)	
> High school	1873 (63.4)	1,691 (56.2)	1,547 (49.2)	
Marital status				<0.001
Married/living with partner	1923 (61.4)	2,363 (68.8)	2,546 (69.4)	
Never married	730 (21.2)	487 (14.5)	410 (11.6)	
Others	677 (17.4)	739 (16.7)	893 (19.0)	
Smoking status				<0.001
Current	733 (23.9)	774 (23.9)	809 (22.0)	
Former	713 (21.8)	940 (25.5)	1,277 (31.3)	
Never	1884 (54.3)	1875 (50.6)	1765 (46.7)	
Alcohol consumption				<0.001
Current	2,402 (77.5)	2,532 (74.7)	2,692 (73.2)	
Former	468 (11.4)	577 (14.4)	649 (15.8)	
Never	460 (11.1)	480 (10.9)	510 (11.1)	
Hypertension				<0.001
Yes	714 (17.5)	1,389 (33.6)	2,362 (56.2)	
No	2,616 (82.5)	2,200 (66.4)	1,489 (43.8)	
All-cause death				<0.001
No	2,871 (90.4)	2,865 (85.8)	2,798 (79.0)	
Yes	459 (9.6)	724 (14.2)	1,053 (21.0)	
Cardiovascular death				<0.001
No	3,194 (97.2)	3,369 (96.2)	3,487 (93.1)	
Yes	136 (2.8)	220 (3.8)	364 (6.9)	

### 3.2 HALP and all-cause mortality in NHANES

**Figure 2:**
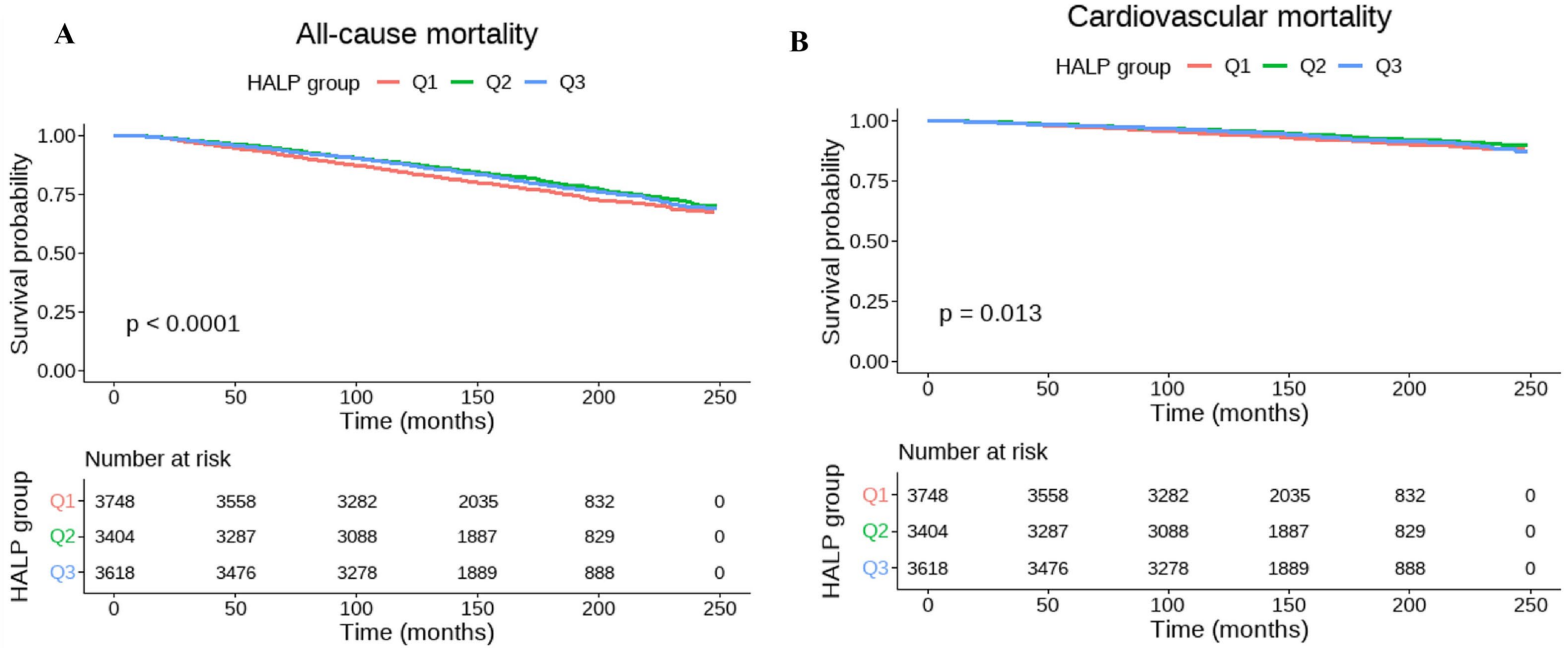
Kaplan–Meier survival curves for all-cause and cardiovascular mortality according to HALP tertiles in NHANES 1999–2010. Panel **A** shows all-cause mortality, and Panel **B** shows cardiovascular mortality. Q1 represents the lowest HALP tertile, Q2 the middle tertile, and Q3 the highest tertile. *p* values were calculated using log-rank tests. HALP, hemoglobin-albumin-lymphocyte-platelet index.

**Figure 3:**
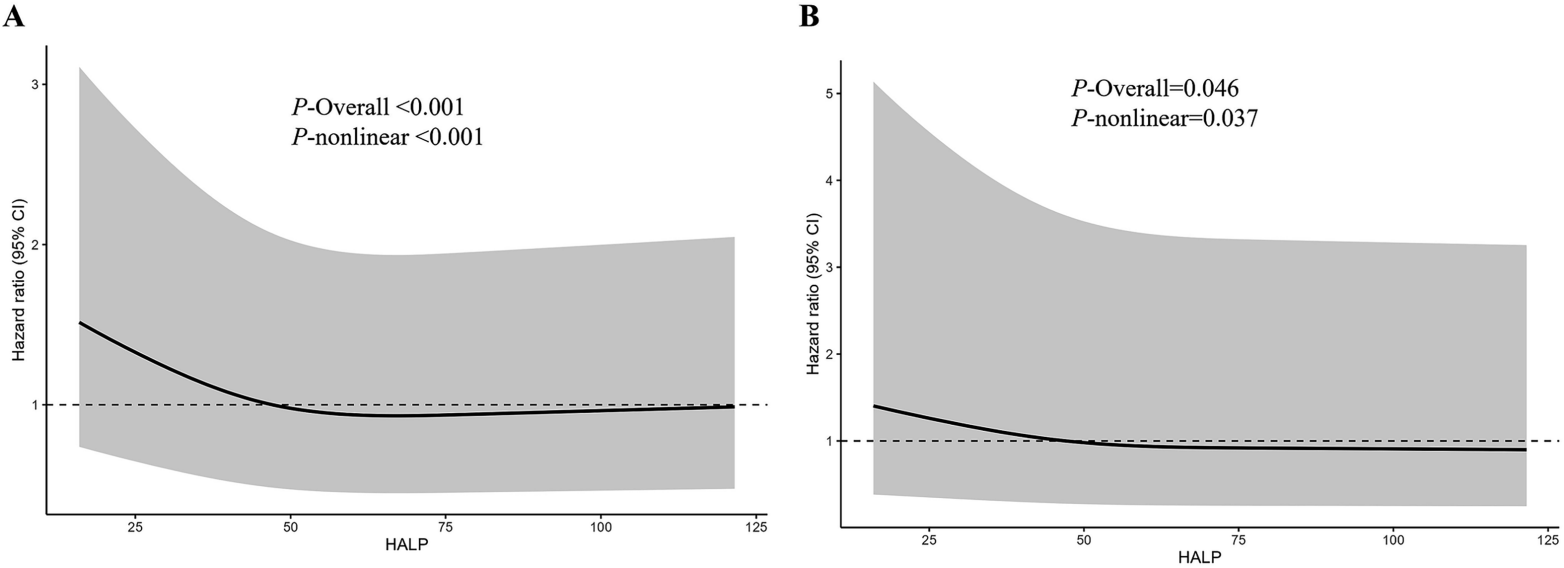
Restricted cubic spline analyses of the association between HALP and mortality in NHANES 1999–2010. Panel **A** shows all-cause mortality, and Panel **B** shows cardiovascular mortality. The solid line represents the adjusted hazard ratio, and the shaded area represents the 95% confidence interval. Models were adjusted for age, sex, race/ethnicity, education, marital status, poverty-income ratio, smoking status, drinking status, hypertension, estimated glomerular filtration rate, and cMetS. HALP, hemoglobin-albumin-lymphocyte-platelet index; cMetS, continuous metabolic syndrome score.

All-cause survival differed markedly across HALP tertiles on Kaplan–Meier analysis, with poorer survival observed in participants with lower HALP levels (log-rank *p* < 0.0001) (Figure 2A). In absolute terms, the estimated 10-year risk of all-cause mortality was highest in the lowest HALP tertile and lower in the middle and highest tertiles (Q1: 15.4, 95% CI 14.2–16.6%; Q2: 11.8, 95% CI 10.8–12.9%; Q3: 12.2, 95% CI 11.1–13.3%; Supplementary Table 1). In survey-weighted Cox models, higher HALP was associated with lower all-cause mortality after adjustment for demographic, socioeconomic, lifestyle, and clinical covariates. Using Q1 as the reference, the adjusted hazard of all-cause death was lower in Q2 (HR 0.78, 95% CI 0.69–0.88) and remained lower in Q3 (HR 0.86, 95% CI 0.77–0.95)(Table 2). Restricted cubic spline analyses supported a significant and nonlinear relationship between HALP and all-cause mortality (*P*-overall < 0.001; *P*-nonlinear < 0.001) (Figure 3A), indicating that the association varied across the HALP distribution rather than changing linearly.

**Table 2:** Associations of HALP tertiles with all-cause mortality and cardiovascular mortality in NHANES 1999–2010.

HALP (tertiles)	Crude		Model 1		Model 2		Model 3	
	HR (95% CI)	*P*	HR (95% CI)	*P*	HR (95% CI)	*P*	HR (95% CI)	*P*
All-cause mortality								
Q1 (lowest)	Reference		Reference		Reference		Reference	
Q2	0.76 (0.67–0.86)	<0.001	0.83 (0.74–0.93)	0.002	0.79 (0.70–0.89)	<0.001	0.78 (0.69–0.88)	<0.001
Q3	0.83 (0.75–0.92)	<0.001	1.00 (0.91–1.11)	0.970	0.89 (0.80–0.98)	0.024	0.86 (0.77–0.95)	0.004
Cardiovascular mortality								
Q1 (lowest)	Reference		Reference		Reference		Reference	
Q2	0.73 (0.57–0.92)	0.008	0.80 (0.63–1.01)	0.064	0.78 (0.62–0.99)	0.042	0.77 (0.61–0.98)	0.036
Q3	0.77 (0.62–0.95)	0.015	0.93 (0.75–1.16)	0.536	0.87 (0.70–1.09)	0.225	0.82 (0.65–1.03)	0.092

### 3.3 HALP and CVD mortality in NHANES

Cardiovascular survival also differed across HALP tertiles (log-rank *p* = 0.013)(Figure 2B). In multivariable survey-weighted Cox regression, Q2 was associated with lower CVD mortality relative to Q1 (HR 0.77, 95% CI 0.61–0.98), whereas Q3 showed a similar direction but did not reach statistical significance (HR 0.82, 95% CI 0.65–1.03)(Table 2). Spline analyses indicated an overall association with evidence of nonlinearity (*P*-overall = 0.046; *P*-nonlinear = 0.037) (Figure 3B).

### 3.4 Joint classification of metabolic burden (cMetS) and HALP

**Figure 4:**
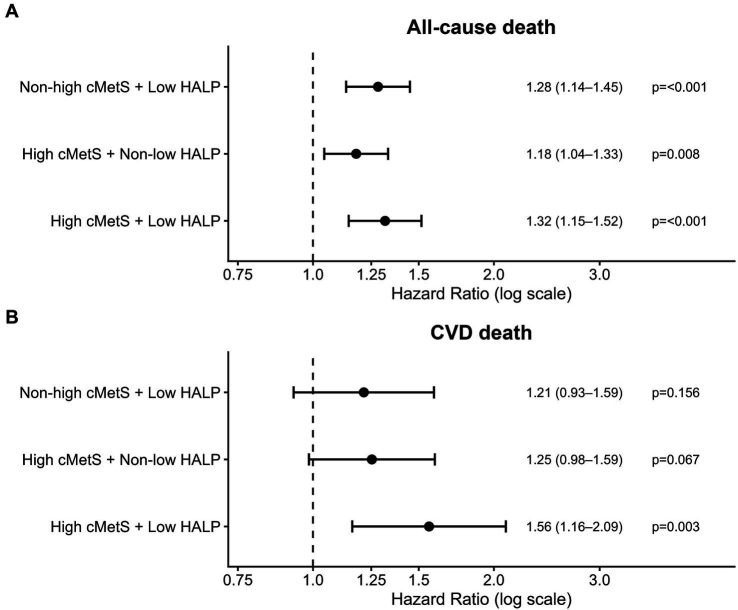
Joint associations of cMetS category and HALP category with all-cause and cardiovascular mortality. Panel **A** shows all-cause mortality, and Panel **B** shows cardiovascular mortality. Hazard ratios and 95% confidence intervals were estimated using survey-weighted Cox proportional hazards models. The reference group was participants with non-high cMetS and non-low HALP. High cMetS was defined as the highest cMetS tertile, and low HALP was defined as the lowest HALP tertile. Models were adjusted for age, sex, race/ethnicity, education, marital status, poverty-income ratio, smoking status, drinking status, hypertension, estimated glomerular filtration rate, and survey cycle. cMetS, continuous metabolic syndrome score; HALP, hemoglobin-albumin-lymphocyte-platelet index.

To evaluate whether HALP provided additional prognostic information beyond metabolic burden, participants were jointly classified into four categories according to cMetS (non-high vs. high) and HALP (non-low vs. low), using the non-high cMetS + non-low HALP group as the reference. Compared with the reference group, elevated risks were mainly observed in strata characterized by low HALP, with the greatest risk in the high cMetS + low HALP category. For all-cause mortality, the high cMetS + low HALP group showed the greatest elevation in risk relative to the reference group (HR 1.32, 95% CI 1.15–1.52; *p* < 0.001)(Figure 4A). For CVD mortality, the same joint category remained most strongly associated with risk (HR 1.56, 95% CI 1.16–2.09; *p* = 0.003), whereas the other joint categories showed weaker or borderline associations (high cMetS + non-low HALP: HR 1.25, 95% CI 0.98–1.59; *p* = 0.067; non-high cMetS + low HALP: HR 1.21, 95% CI 0.93–1.59; *p* = 0.156)(Figure 4B).

No statistically significant interaction was observed between HALP and cMetS for either all-cause or CVD mortality (*P* for interaction = 0.73 and 0.89, respectively). The inverse association between HALP and mortality remained directionally consistent across different levels of metabolic burden, indicating no evidence of effect modification by cardiometabolic risk status.

### 3.5 Incremental prognostic value of HALP beyond cMetS

To evaluate whether HALP provided incremental prognostic information beyond cMetS, we compared two nested models based on the fully adjusted covariate set. Model A included the fully adjusted covariates and cMetS, whereas Model B additionally incorporated HALP modeled flexibly to account for its nonlinear association with mortality. For all-cause mortality, adding HALP was associated with a modest increase in Harrell’s C statistic (Model A: 0.8666; Model B: 0.8685; ΔC = 0.0018). Bootstrap resampling supported a small improvement in discrimination (95% percentile CI: 0.0010–0.0036; 95% basic CI: 0.0001–0.0027). Decision curve analysis for 10-year all-cause mortality suggested a modest net benefit advantage for the model including HALP across low-to-moderate threshold probabilities (Supplementary Figure 1). When HALP was entered as tertiles or as a winsorized linear term, the increment in discrimination was smaller (Supplementary Table 2).

### 3.6 Sensitivity analyses

To minimize potential reverse causality, analyses were repeated after excluding participants with follow-up < 24 months. Associations between HALP tertiles and mortality outcomes remained largely consistent with the primary findings. Compared with Q1, higher HALP was associated with a lower risk of all-cause mortality (Q2 vs. Q1: HR 0.79, 95% CI 0.69–0.90; Q3 vs. Q1: HR 0.87, 95% CI 0.76–1.00). Similar patterns were observed for CVD mortality, with a significantly lower risk in Q2 (HR 0.78, 95% CI 0.62–0.99), whereas Q3 remained directionally protective but not statistically significant (HR 0.84, 95% CI 0.66–1.07). Overall, these sensitivity analyses supported the robustness of the main results (Supplementary Table 3).

In the primary cause-specific Cox analysis, lower HALP was associated with higher CVD mortality, although the association was less consistent than that observed for all-cause mortality. By contrast, when non-CVD death was treated as a competing event in Fine-Gray models, the corresponding subdistribution hazard ratios were attenuated and no longer statistically significant (Q2 vs. Q1: sHR 0.87, 95% CI: 0.70–1.10, *p* = 0.246; Q3 vs. Q1: sHR 0.92, 95% CI: 0.73–1.17,*p* = 0.509; Supplementary Table 4). This discrepancy indicates that the association between HALP and CVD mortality was weaker when the cumulative incidence of CVD mortality was evaluated in the presence of competing non-CVD mortality.

### 3.7 Supportive findings from the hospital-based external cohort

In the hospital-based external cohort (*n* = 500), baseline characteristics varied across cMetS tertiles (Supplementary Table 5). Participants with higher cMetS were older and had a higher prevalence of hypertension. The proportion of all-cause deaths increased across increasing cMetS tertiles, whereas cardiovascular deaths were infrequent overall. Only 36 all-cause deaths and 17 cardiovascular deaths occurred during follow-up, therefore, findings from this cohort, particularly for cardiovascular mortality, should be interpreted as supportive and exploratory rather than definitive validation. When participants were stratified by HALP tertiles, Kaplan–Meier curves showed significant separation for all-cause mortality (log-rank *p* = 0.024), with the lowest HALP group exhibiting the poorest survival (Supplementary Figure 2). In multivariable Cox models (T1 as reference), higher HALP tertiles were independently associated with a lower risk of all-cause mortality (T2: HR 0.275, 95% CI: 0.095–0.792, *p* = 0.017; T3: HR 0.196, 95% CI: 0.041–0.939, *p* = 0.042). In contrast, no significant association was observed between HALP tertiles and CVD mortality (log-rank *p* = 0.258; adjusted T2: HR 0.983, 95% CI: 0.241–4.006, *p* = 0.981; T3: HR 0.745, 95% CI: 0.080–6.915, *p* = 0.796) (Supplementary Table 6), with wide confidence intervals likely reflecting the limited number of cardiovascular deaths in the validation cohort.

## 4 Discussion

In this analysis of U.S. adults from NHANES with mortality follow up, we examined the associations of the HALP index with all-cause and CVD mortality across the continuum of cardiometabolic risk quantified by cMetS. The results indicate that the HALP index is inversely associated with mortality in a nationally representative sample of United States adults across a wide range of cardiometabolic profiles quantified by cMetS. Higher HALP was associated with lower all-cause mortality, and spline analyses suggested a nonlinear relationship. For CVD mortality, the association was directionally similar but less consistent. In joint analyses, participants with higher cMetS and low HALP had the highest risks of both all-cause and CVD mortality. No statistically significant interaction was observed between HALP and cMetS for either all-cause or CVD mortality. Findings from the supportive external cohort were consistent with the all-cause mortality association, whereas CVD mortality estimates were less stable because of the limited number of cardiovascular deaths.

The inverse association between HALP and all-cause mortality is biologically plausible given the components of HALP. Hemoglobin reflects oxygen-carrying capacity and anemia-related reserve, albumin is influenced by inflammatory burden and nutritional status, lymphocyte count relates to immune competence and chronic stress physiology, and platelet count reflects inflammatory and thrombotic activity (17). These domains are relevant to long-term survival in metabolically dysregulated states, where chronic low-grade inflammation, endothelial dysfunction, and multi-organ vulnerability are prevalent and may magnify the impact of reduced physiologic reserve (18). Prior work in coronary heart disease, heart failure, acute myocardial infarction, and diabetes cohorts has associated lower HALP with higher mortality, suggesting that HALP may capture prognostic information beyond conventional cardiometabolic risk factors (19, 20). Compared with disease-selected cohorts, our work adds a distinct contribution by placing HALP within a continuous cMetS framework, which more directly reflects the graded nature of metabolic dysregulation and helps interpret heterogeneity in prognosis across the cardiometabolic spectrum.

An important reason for evaluating HALP in the present study is that it is derived entirely from routine laboratory measures that are inexpensive and widely obtainable, even though it is not yet a broadly established clinical risk score. In this context, the value of HALP lies less in its current status as a formal clinical instrument and more in its ability to integrate information on anemia-related reserve, nutritional status, immune competence, and thrombo-inflammatory activity within a single composite index. Because these dimensions are not fully captured by cMetS, HALP may help characterize systemic vulnerability that contributes to heterogeneity in mortality risk across the cardiometabolic spectrum.

The restricted cubic spline analyses suggested a nonlinear, L-shaped association between HALP and mortality risk. The risk was markedly elevated at very low HALP values and declined steeply with increasing HALP in the lower range, followed by a more gradual decrease across higher HALP levels. Such a pattern is biologically plausible because extremely low HALP may reflect the co-occurrence of anemia, hypoalbuminemia, immune dysregulation, and platelet-related inflammatory activation, which together indicate reduced physiologic reserve and heightened vulnerability. Similar nonlinear patterns have been reported when HALP was evaluated in other cardiometabolic populations, supporting the view that the prognostic gradient of HALP is strongest at low levels (19, 21). Notably, the confidence intervals widened at the extremes of HALP, which is a recognized feature of flexible modeling approaches such as restricted cubic splines when data are sparse at the tails (22). Therefore, inferences should focus primarily on the range with adequate observations.

A central contribution of this study is the joint evaluation of HALP and cMetS, which clarifies how HALP relates to mortality risk across a continuous spectrum of metabolic dysregulation rather than in isolation. The observation that participants with higher cMetS and lower HALP had the highest mortality risk supports the interpretability of the stratified and joint findings. cMetS captures the overall degree of metabolic dysregulation, whereas HALP summarizes hematologic and immune–nutritional status. Considering these measures together may therefore help identify individuals who are both metabolically high risk and have an unfavorable systemic profile, in whom mortality risk is most concentrated. Notably, the absence of a statistically significant HALP×cMetS interaction does not contradict the joint-exposure results. Joint categories emphasize differences in absolute risk across combined exposure groups, whereas interaction testing assesses whether the relative association differs by cMetS strata, the latter can be underpowered when exposures are categorized and when cardiovascular deaths are limited. Accordingly, these findings are best interpreted as providing no evidence of multiplicative interaction while still supporting clinically meaningful risk characterization using the combined assessment of HALP and cMetS.

An important additional finding is that HALP provided only a modest increment in model discrimination beyond cMetS and other established covariates, and this increment became evident primarily when HALP was modeled flexibly rather than as a simple linear term. This is consistent with the nonlinear L-shaped association observed in the primary analyses, suggesting that HALP may not act as a uniformly linear prognostic marker across its full distribution. Accordingly, HALP should be interpreted less as a strong standalone enhancer of mortality prediction and more as a complementary marker of systemic vulnerability whose prognostic contribution is detectable but limited in magnitude.

Previous studies have shown that metabolic risk staging can be further refined by incorporating markers that reflect inflammatory and nutritional status. In an NHANES-based study of participants with CKM stages 0–3, stress hyperglycemia ratio was associated with all-cause mortality, and additional analyses incorporating RDW, albumin, and the RDW-to-albumin ratio further supported the relevance of inflammatory-nutritional status for risk characterization within metabolic staging (23).

Complementary evidence from cMetS-based epidemiologic modeling further supports the close coupling between metabolic dysregulation and inflammation: mechanistic analyses have incorporated hs-CRP and IL-6 when quantifying pathways related to cardiometabolic risk and cMetS, reinforcing that inflammatory activity tracks with cMetS-defined metabolic burden even when mortality is not the primary endpoint (24, 25). Collectively, these studies establish a clear rationale for integrating systemic biomarkers with cMetS. Our findings extend this paradigm by demonstrating that the joint assessment of HALP and metabolic burden aligns conceptually with prior models that combine metabolic risk with inflammatory and nutritional status to better capture prognostic heterogeneity. Emerging evidence suggests that myocardial strain imaging may detect subclinical cardiac dysfunction before overt structural abnormalities become apparent. In this context, HALP and myocardial strain may reflect related but distinct dimensions of vulnerability, with HALP capturing systemic nutritional, inflammatory, immune, and hematologic dysregulation, and myocardial strain reflecting downstream myocardial functional impairment. Future studies should evaluate whether combining HALP with myocardial strain parameters could further refine risk characterization in metabolically high-risk populations (26).

For CVD mortality, we observed directionally similar but less consistent associations, and the supportive external cohort results were less stable. This pattern is plausible because cardiovascular death is less frequent than all-cause mortality, resulting in fewer events, reduced statistical power, and wider confidence intervals. In addition, CVD mortality is susceptible to competing risks from non-cardiovascular death and to potential misclassification of cause of death, both of which may attenuate associations and increase variability, particularly when endpoint ascertainment differs across cohorts (27). Under a competing-risk framework, non-cardiovascular death can preclude the occurrence of cardiovascular death; thus, associations observed in cause-specific Cox models may be attenuated in Fine–Gray models that quantify cumulative incidence in the presence of competing events. Accordingly, the HALP-CVD mortality association should be interpreted cautiously after accounting for competing risks. In this context, we treated the cause-specific Cox model as the primary analysis for CVD mortality because it is better suited to evaluating the etiologic association between HALP and CVD death. By contrast, the Fine-Gray model addresses a different question, namely whether HALP is associated with the cumulative incidence of CVD mortality when non-CVD death is considered a competing event. The attenuation of the association in the Fine-Gray analysis suggests that part of the prognostic information captured by HALP may reflect broader systemic vulnerability that influences multiple causes of death, rather than CVD death alone. Clinically, this means that HALP appears more robust as a marker of overall mortality risk than as a marker of cardiovascular-specific mortality after accounting for competing non-CVD death. Consistent with this interpretation, an NHANES-based study in U. S. adults with type 2 diabetes likewise reported more stable associations for all-cause mortality than for CVD mortality,illustrating that cause-specific mortality signals may be less robust when event numbers are limited and competing risks are present (28).

Several limitations should be considered. First, as an observational analysis, residual confounding cannot be excluded despite multivariable adjustment. Second, HALP and cMetS were assessed at baseline, and changes over time were not captured; subclinical illness may also influence albumin and blood cell counts, so some reverse causality may persist despite excluding early follow up and conducting sensitivity analyses. Third, the supportive external cohort included only 36 all-cause deaths and 17 cardiovascular deaths, limiting the precision of effect estimates, particularly for cause-specific analyses. Therefore, the external results should be interpreted primarily as supportive for all-cause mortality rather than as definitive validation of cardiovascular mortality. In addition, cardiovascular death in the external cohort was classified from hospital records and telephone follow-up without autopsy-based morphological confirmation, which may have introduced outcome misclassification.

## 5 Conclusion

In summary, higher HALP was independently and nonlinearly associated with lower all-cause mortality across the cMetS spectrum. Participants with high metabolic burden and low HALP had the highest risks of all-cause and cardiovascular mortality. HALP also provided modest additional prognostic information beyond cMetS, although the magnitude of improvement was limited. These findings support HALP as an inexpensive and readily available complementary marker of systemic vulnerability rather than a major standalone predictor. Findings from the supportive external cohort were directionally consistent with the all-cause mortality association, whereas the cardiovascular mortality findings should be interpreted cautiously because of limited event numbers and less rigorous endpoint ascertainment.

## Data Availability

The original contributions presented in the study are included in the article/Supplementary material, further inquiries can be directed to the corresponding author.

## Supplementary material

SUPPLEMENTARY FIGURE 1 Decision curve analysis comparing models with and without HALP for 10-year all-cause mortality. Model A included age, sex, race/ethnicity, education, marital status, poverty-income ratio, smoking status, drinking status, hypertension, estimated glomerular filtration rate, survey cycle, and cMetS. Model B included all variables in Model A plus HALP modeled flexibly. The model including HALP showed modestly greater net benefit across low-to-moderate threshold probabilities.

SUPPLEMENTARY FIGURE 2 Kaplan–Meier survival curves according to HALP tertiles in the hospital-based external cohort. Panel A shows all-cause mortality, and Panel B shows cardiovascular mortality. T1 represents the lowest HALP tertile, T2 the middle tertile, and T3 the highest tertile. Findings from this cohort should be interpreted as supportive and exploratory because of the limited number of outcome events, particularly cardiovascular deaths. HALP, hemoglobin-albumin-lymphocyte-platelet index.
