# Supplementary figures and images for: HALP, a routine nutrition-inflammation index, and mortality across the cMetS spectrum: NHANES with supportive external cohort evidence
Source: Front Nutr. 2026 May 20;13:1818651. doi: 10.3389/fnut.2026.1818651 (PMC13234567; doi:10.3389/fnut.2026.1818651)

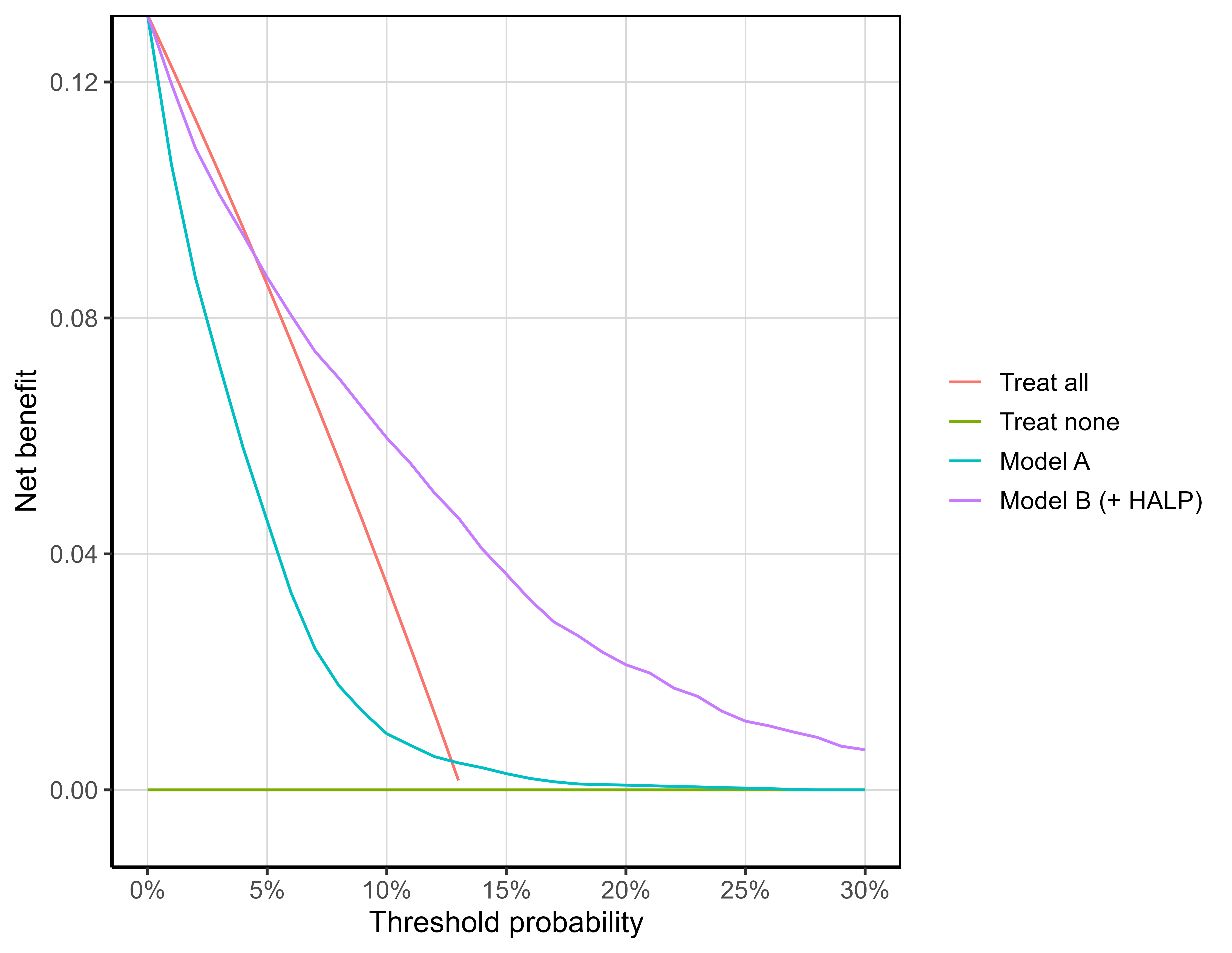

Supplement: SUPPLEMENTARY FIGURE 1 — Decision curve analysis comparing models with and without HALP for 10-year all-cause mortality. Model A included age, sex, race/ethnicity, education, marital status, poverty-income ratio, smoking status, drinking status, hypertension, estimated glomerular filtration rate, survey cycle, and cMetS. Model B included all variables in Model A plus HALP modeled flexibly. The model including HALP showed modestly greater net benefit across low-to-moderate threshold probabilities. [file Image_1.PNG]

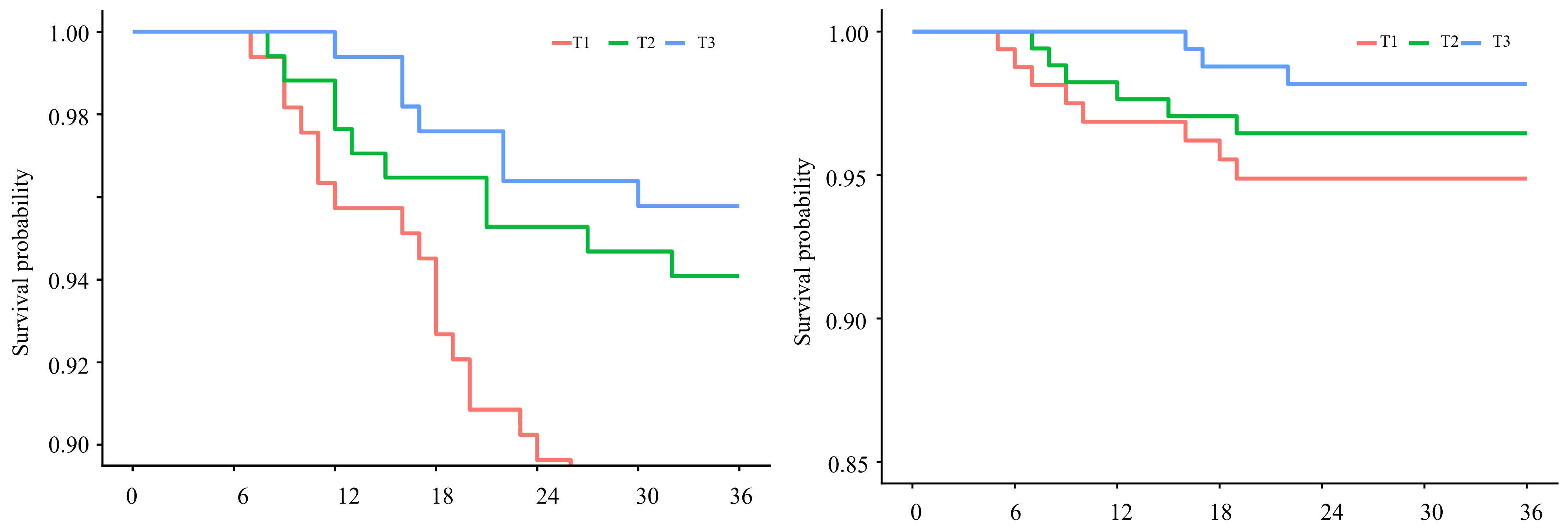

Supplement: SUPPLEMENTARY FIGURE 2 — Kaplan–Meier survival curves according to HALP tertiles in the hospital-based external cohort. Panel A shows all-cause mortality, and Panel B shows cardiovascular mortality. T1 represents the lowest HALP tertile, T2 the middle tertile, and T3 the highest tertile. Findings from this cohort should be interpreted as supportive and exploratory because of the limited number of outcome events, particularly cardiovascular deaths. HALP, hemoglobin-albumin-lymphocyte-platelet index. [file Image_2.JPEG]
