# Supplementary material for: HALP, a routine nutrition-inflammation index, and mortality across the cMetS spectrum: NHANES with supportive external cohort evidence
Source: Front Nutr. 2026 May 20;13:1818651. doi: 10.3389/fnut.2026.1818651 (PMC13234567; doi:10.3389/fnut.2026.1818651)
Supplement: Supplementary file 3 [file Table_1.doc]

Supplementary Table 1. Estimated 10-year absolute risk of all-cause mortality across HALP tertiles

| HALP tertile | 10-year survival (%) | 10-year absolute risk (%) | 95% CI for absolute risk (%) |
| --- | --- | --- | --- |
| Q1 (lowest) | 84.6 | 15.4 | 14.2–16.6 |
| Q2 | 88.2 | 11.8 | 10.8–12.9 |
| Q3 (highest) | 87.8 | 12.2 | 11.1–13.3 |

**Note:** The 10-year absolute risk estimates were derived from Kaplan–Meier survival estimates at 120 months.
