# Supplementary material for: HALP, a routine nutrition-inflammation index, and mortality across the cMetS spectrum: NHANES with supportive external cohort evidence
Source: Front Nutr. 2026 May 20;13:1818651. doi: 10.3389/fnut.2026.1818651 (PMC13234567; doi:10.3389/fnut.2026.1818651)
Supplement: Supplementary file 4 [file Table_2.doc]

Supplementary Table 2. Incremental discrimination analysis for all-cause mortality beyond cMetS

| Outcome | Model | Description | Harrell’s C | ΔC vs Model A | 95% CI | *P* value |
| --- | --- | --- | --- | --- | --- | --- |
| All-cause mortality | Model A | Fully adjusted covariates+ cMetS | 0.8666 | Ref | — | — |
| All-cause mortality | Model B | Model A + HALP | 0.8685 | 0.0018 | 0.0010-0.0036 | < 0.001 |

Note: The 95% CI shown for Model B is the percentile bootstrap confidence interval. The corresponding basic bootstrap 95% CI was 0.0001–0.0027. When HALP was modeled as tertiles or as a winsorized linear term, the increment in Harrell’s C was smaller; therefore, these alternative specifications were not included in the main supplementary table.
