# Supplementary material for: HALP, a routine nutrition-inflammation index, and mortality across the cMetS spectrum: NHANES with supportive external cohort evidence
Source: Front Nutr. 2026 May 20;13:1818651. doi: 10.3389/fnut.2026.1818651 (PMC13234567; doi:10.3389/fnut.2026.1818651)
Supplement: Supplementary file 5 [file Table_3.docx]

# Supplementary Table 3. Sensitivity analyses of the associations between HALP tertiles and mortality after excluding participants with follow-up < 24 months.

| Outcome | HALP (Tertiles) | HR (95% CI) | *P* value |
| --- | --- | --- | --- |
| All-cause mortality | Q2 vs Q1 | 0.79(0.69-0.90) | <0.001 |
|  | Q3 vs Q1 | 0.87(0.76-1.0) | 0.0448 |
| CVD mortality | Q2 vs Q1 | 0.78(0.62-0.99) | 0.037 |
|  | Q3 vs Q1 | 0.84(0.66-1.07) | 0.156 |

Note: Q1 (lowest) served as the reference. Estimates were derived from survey-weighted Cox models adjusted for the same covariates as Model 3 in Table 2.
