# Supplementary material for: HALP, a routine nutrition-inflammation index, and mortality across the cMetS spectrum: NHANES with supportive external cohort evidence
Source: Front Nutr. 2026 May 20;13:1818651. doi: 10.3389/fnut.2026.1818651 (PMC13234567; doi:10.3389/fnut.2026.1818651)
Supplement: Supplementary file 6 [file Table_4.docx]

Supplementary Table 4. Fine-Gray competing-risk models for cardiovascular mortality according to HALP tertiles in NHANES 1999-2010

| HALP tertiles | Fine–Gray sHR (95% CI) | *P* value |
| --- | --- | --- |
| Q2 vs Q1 | 0.87 (0.70-1.10) | 0.246 |
| Q3 vs Q1 | 0.92 (0.73-1.17) | 0.509 |

Abbreviations: sHR, subdistribution hazard ratio; CI, confidence interval; SD, standard deviation. Models were adjusted for age, sex, race, family income-to-poverty ratio, education, marital status, drinking, smoking, hypertension category, estimated glomerular filtration rate, and cMetS.
