# Supplementary material for: HALP, a routine nutrition-inflammation index, and mortality across the cMetS spectrum: NHANES with supportive external cohort evidence
Source: Front Nutr. 2026 May 20;13:1818651. doi: 10.3389/fnut.2026.1818651 (PMC13234567; doi:10.3389/fnut.2026.1818651)
Supplement: Supplementary file 7 [file Table_5.docx]

Supplementary Table 5. Baseline characteristics according to cMetS tertiles in the hospital-based external cohort.

| Variable | cMetS T1 | cMetS T2 | cMetS T3 | *P* |
| --- | --- | --- | --- | --- |
| N | 167 | 167 | 166 |  |
| Age,year | 60.00 (57.00, 64.00) | 68.00 (65.00, 71.00) | 76.00 (74.00, 78.00) | <0.001 |
| HALP | 42.70 (40.30, 45.30) | 47.50 (43.95, 51.00) | 49.55 (44.12, 55.30) | <0.001 |
| Sex |  |  |  | <0.001 |
| Male | 77 (46.1) | 73 (43.7) | 85 (51.2) |  |
| Female | 90 (53.9) | 94 (56.3) | 81 (48.8) |  |
| Hypertension, yes n (%) | 19 (11.4) | 45 (26.9) | 73 (44.0) | <0.001 |
| All-cause mortality, events n (%) | 7 (4.2) | 10 (6.0) | 19 (11.4) | 0.031 |
| Cardiovascular mortality, events n (%) | 3 (1.8) | 6 (3.6) | 8 (4.8) | 0.319 |

Data are presented as median (interquartile range) for continuous variables and n (%) for categorical variables. cMetS tertiles were defined using cohort-specific tertile cutpoints in the hospital-based external cohort. T1, T2, and T3 represent the lowest, middle, and highest cMetS tertiles, respectively. P values were calculated using the Kruskal–Wallis test for continuous variables and chi-square or Fisher’s exact test for categorical variables, as appropriate. cMetS, continuous metabolic syndrome score; HALP, hemoglobin-albumin-lymphocyte-platelet index.
