# Supplementary material for: HALP, a routine nutrition-inflammation index, and mortality across the cMetS spectrum: NHANES with supportive external cohort evidence
Source: Front Nutr. 2026 May 20;13:1818651. doi: 10.3389/fnut.2026.1818651 (PMC13234567; doi:10.3389/fnut.2026.1818651)
Supplement: Supplementary file 8 [file Table_6.doc]

## Supplementary Table 6. Association between HALP tertiles and mortality in the hospital-based external cohort

| All-cause mortality HR (95% CI) | **Model 1** | *P* | **Model 2** | *P* |
| --- | --- | --- | --- | --- |
| T1 (Low) | 1.00 (ref) | — | 1.00 (ref) | — |
| T2 (Middle) | 0.257 (0.090-0.729) | 0.011 | 0.275 (0.095-0.792) | 0.017 |
| T3 (High) | 0.172 (0.036-0.811) | 0.026 | 0.196 (0.041-0.939) | 0.042 |
| CVD mortality HR (95% CI) |  |  |  |  |
| T1 (Low) | 1.00 (ref) | — | 1.00 (ref) | — |
| T2 (Middle) | 0.786 (0.201-3.074) | 0.729 | 0.983 (0.241-4.006) | 0.981 |
| T3 (High) | 0.491 (0.055-4.402) | 0.525 | 0.745 (0.080-6.915) | 0.796 |
